# Bortezomib-Induced Reversible Cardiomyopathy: Recovered With Guideline-Directed Medical Therapy

**DOI:** 10.7759/cureus.20295

**Published:** 2021-12-09

**Authors:** Swethika H Sundaravel, Rosalyn I Marar, Muhannad A Abbasi, Muhamed Baljevic, Jeremy R Stone

**Affiliations:** 1 Cardiology, University of Nebraska Medical Center, Omaha, USA; 2 Internal Medicine, University of Nebraska Medical Center, Omaha, USA; 3 Oncology, University of Nebraska Medical Center, Omaha, USA

**Keywords:** cardio-oncology, gdmt, reversible, cardiomyopathy, bortezomib

## Abstract

Bortezomib (BTZ) is a proteasome inhibitor (PI) used for the treatment of several hematologic malignancies, including multiple myeloma (MM), and various lymphomas including mantle cell lymphoma (MCL). It acts via disruption of the ubiquitin-proteasome pathway which plays a major role in regulating cell cycle and inhibiting synthesis of nuclear factor kappa-light-chain-enhancer of activated B cells (NF-KB). The ubiquitin-proteasome pathway is also important in maintaining the integral signaling in cardiac myocytes. By inhibiting this system, BTZ induces cellular apoptosis in cancer cells, and possibly the cardiomyocytes. BTZ-induced cardiotoxicity in monotherapy and combination treatments is not well described in the literature.

We observed a series of three patients who developed cardiotoxicity after treatment with BTZ. All patients had echocardiograms every 3 months until recovery to assess ejection fraction (EF) and global longitudinal strain (GLS). Two of the patients had a cardiac MRI (CMR) conducted during follow-up to assess for late gadolinium enhancement (LGE).

The median age of our patients was 55 years (range 37-74). Two of them had MM, while one patient had MCL. Table 1 demonstrates patient demographics, past medical histories, and the cumulative dose and duration of BTZ therapy. Of the three patients, only one had a heart failure exacerbation at diagnosis. The other two patients were diagnosed with asymptomatic left ventricular systolic dysfunction on routine pre-transplant echocardiograms. Most importantly, all three patients had improvement or normalization of cardiac function with discontinuation of BTZ and initiation of guideline-directed medical therapy (GDMT) for heart failure. The median duration to recovery was 5 months (range 3-13). One patient had underlying non-compaction cardiomyopathy, and although EF did not normalize, it recovered to his previous baseline. All 3 patients had improvement in GLS. Two patients underwent CMRI at the time of cardiomyopathy diagnosis and neither of them had any late gadolinium enhancement. Since there was no routine pre-treatment echocardiogram, using the GLS trend to detect subclinical cardiac dysfunction was not possible.

This case series demonstrates that BTZ-induced cardiomyopathy is potentially reversible with discontinuation of the drug and early initiation of GDMT. Further studies are needed to determine the ideal surveillance strategy for BTZ-induced cardiomyopathy.

## Introduction

Multiple myeloma (MM) is a widely prevalent hematological malignancy that affects thousands of people worldwide, with a median age of 65-74 years at diagnosis [1, 2]. The incidence rate of MM is reported to be 2.1 per 100,000 persons in 2016 [3]. Myeloma patients are at an increased risk of developing cardiovascular events, which can be attributed to the direct toxicity of light chain accumulation, deposition of light chains in myocytes causing Light Chain Amyloidosis, or increased thrombotic risk causing coronary events and/or venous thromboembolism [4]. The incidence of cardiovascular events in MM patients is variable and depends on multiple factors such as patient demographics, disease severity, and involvement of other organs. In addition, there is also an increased risk of developing cardiovascular complications while receiving standard chemo/immunotherapy during MM treatment [5-7].

Proteasome inhibitors (PIs) are a recent addition to the armamentarium of cancer chemotherapeutics. They are widely used for several malignancies, including MM and lymphoma. The first PI approved for the treatment of MM was BTZ in 2003. Subsequently, there have been few other PIs that have been approved for the treatment of refractory hematological malignancies [8].

PIs are known culprits of cardiovascular toxicity, carfilzomib more often than bortezomib (BTZ) (7% versus 2-5% respectively) [9]. Carfilzomib is thought to be more commonly associated with new-onset or progression of pre-existing congestive heart failure (CHF) and myocardial ischemia. BTZ is not consistently associated with a significantly increased risk of cardiotoxicity [10], potentially because it is less studied than Carfilzomib in prospective analyses.

According to data from the phase II SUMMIT trial [11], the Food and Drug Administration approved BTZ in 2003. Partially reversible neurotoxicity was reported as the main non-hematologic side effect. The other main side effects reported in the study include thrombocytopenia and gastrointestinal effects. However, cardiovascular side effects were not mentioned in this trial. There have been isolated case reports of BTZ induced left ventricular (LV) dysfunction and some cases of tachyarrhythmias and conduction abnormalities reported in the literature. However, there is no specific data on the treatment and reversibility of BTZ induced LV dysfunction.

Here, we describe a case series of 3 patients who had BTZ-induced left ventricular dysfunction that recovered with standard guideline-directed medical therapy (GDMT) for heart failure and discontinuation of BTZ, as well as provides a review of the current literature on the topic of BTZ-related cardiotoxicity.

## Case presentation

Patient 1

A 55-year-old man with known non-compaction cardiomyopathy with a baseline left ventricular ejection fraction (LVEF) of 45-50% developed revised international staging system stage II, ultra-high-risk t(4;14) and 1q21.3 MM. He was treated with subcutaneous BTZ, lenalidomide, and dexamethasone in 21-day cycles. BTZ was delivered at 1.3 mg/m^2^ on days 1, 4, 8, and 11 for 4 months (cumulative dose of 20.2mg/m^2^). During follow-up, the patient was experiencing shortness of breath with exertion concerning for worsening CHF. Thereafter, a transthoracic echocardiogram was performed, which showed a new reduction in LVEF (30-35%). Cardiac MRI showed an LVEF of 34% with no late gadolinium enhancement (LGE). Subsequently, BTZ was stopped, and he was started on metoprolol succinate and lisinopril with improvement in LVEF. Following melphalan dose-reduced autologous hematopoietic stem cell transplant (AHSCT), his subsequent therapy included a switch to an alternative PI, ixazomib, for the remainder of consolidative combination therapy for his ultra-high-risk MM.

Patient 2

A 74-year-old man with a history of hypertension developed stage IIB mantle cell lymphoma (MCL). He was started on subcutaneous BTZ, lenalidomide, and dexamethasone in 28-day cycles, with BTZ given weekly. He received 10 months of combination therapy (cumulative BTZ dose of 20.8mg/m^2^). Subsequently, he developed decompensated CHF (LVEF of 40%) requiring hospital admission for intravenous diuresis. He had no previous history of CHF or coronary artery disease, and his prior baseline LVEF was normal. Subsequent CMRI showed an LVEF of 45% with no LGE and BTZ was stopped. Metoprolol succinate and lisinopril were initiated and LVEF improved to 65% over the next 13 months with no further CHF hospitalizations. He achieved a complete response to MCL treatment, but was later diagnosed with MM as a second malignancy for which he was treated with an alternative PI-based (ixazomib) therapy to avoid further BTZ-suspected cardiotoxicity.

Patient 3

A 37-year-old woman with a history of revised international staging system stage II, high-risk MM with extramedullary splenic involvement, received subcutaneous BTZ, lenalidomide, and dexamethasone in 21-day cycles. BTZ was delivered at 1.3 mg/m^2^ on days 1, 4, 8, and 11 for 6 cycles during induction, and then subsequently weekly for consolidation after melphalan200 AHSCT. Her LVEF was preserved after induction therapy and presumably following the melphalan 200 mg/m^2^ pre-AHSCT conditioning. However, after 20 months of weekly BTZ consolidation therapy post-AHSCT, she was found to have a reduced LVEF (45%) on routine echocardiogram performed as part of stem cell transplant evaluation. She had no symptoms of heart failure. By that point, she had received a cumulative BTZ dose of 135 mg/m^2^. Subsequently, BTZ was stopped and metoprolol succinate and lisinopril were initiated. Her LVEF recovered to normal after 3 months of GDMT with no admissions for decompensated CHF. For salvage therapy, she was started on a regimen that was non-PI-containing. However, with disease progression, she was subsequently treated with another PI (carfilzomib)-containing regimen on which she did not experience further reductions in LVEF. 

**Figure 1:**
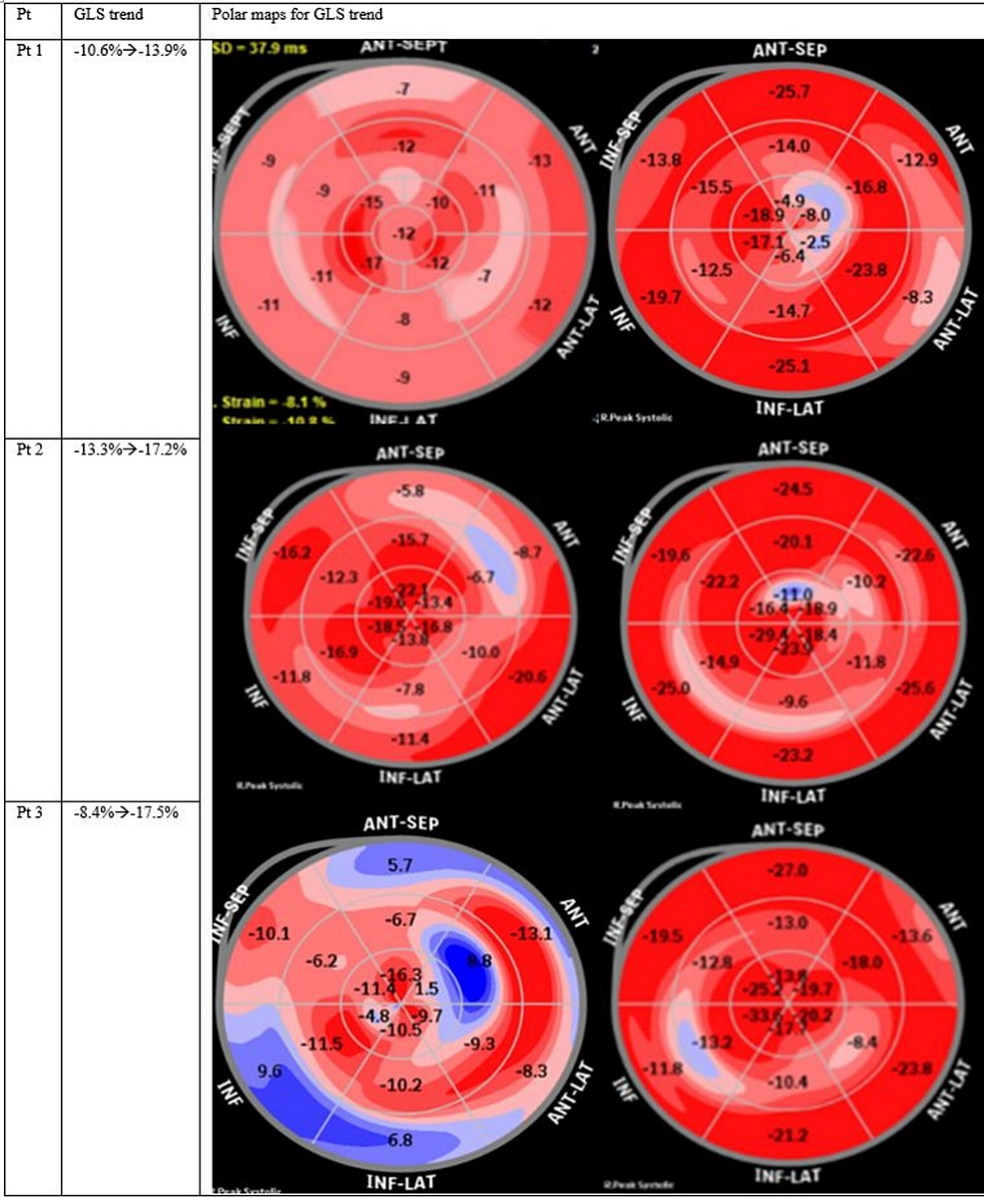
Trend of Global Longitudinal Strain derived by speckle tracking echocardiography. Pt: Patient; GLS: Global Longitudinal Strain

Table 1 demonstrates patient demographics, past medical histories, and the cumulative dose and duration of BTZ therapy. Figure 1 demonstrates the trend of global longitudinal strain pre-and post GDMT.

**Table 1:** Patient demographics, diagnostic studies, and management. HTN: Hypertension; Mel200: High-dose melphalan; VRd: Bortezomib, Lenalidomide, and Dexamethasone; AHSCT: Autologous hematopoietic stem cell transplant; GDMT: guideline-directed medical therapy; NYHA: New York Heart Failure Association; BB: Beta-blocker; ACEi: Angiotensin-Converting Enzyme Inhibitors

Demographics	Patient 1	Patient 2	Patient 3
Age	55	74	37
Sex	Male	Male	Female
Ethnicity	Caucasian	Caucasian	Caucasian
Risk factors	HTN, non-compaction	HTN	Mel200 AHSCT
Type of malignancy	Multiple myeloma	Mantle cell lymphoma	Multiple myeloma
Prior chemotherapy			Mel200
Chemotherapy regimen	VRd	VRd	VRd
Cumulative dose of BTZ (mg/m^2^)	20.2	20.8	135
Duration of BTZ therapy (months)	4	10	27
LVEF trend	35% => 45%	40% => 65%	45% => 60%
GLS trend	-10.6% => -13.9%	-13.3% => -17.2%	-8.4% => -17.5%
Cardiac MRI	34% EF No LGE	45% EF No LGE	n/a
Duration to recovery (months)	5	13	3
GDMT	BB, ACEi	BB, ACEi	BB, ACEi
NYHA class on diagnosis	2	3	1

## Discussion

PIs work by interrupting the Ubiquitin-Proteasome System (UPS), [12] which plays a central role in the degradation and turnover of proteins in human cells. The proteasome system is crucial to maintain cellular homeostasis by regulating intracellular proteolysis of abnormal proteins. In cancer cells, the inhibition of this UPS leads to intracellular accumulation of abnormal proteins, which then triggers a downstream apoptotic cascade leading to the death of cancer cells [13]. Unfortunately, the mechanism of PI-induced cardiotoxicity is poorly understood. Many hypotheses have been proposed to explain this clinical phenomenon. Cardiomyocytes are non-proliferating cells and rely heavily on proteasome-dependent protein turnover. Inhibition of the UPS leads to the accumulation of incompatible cytotoxic proteins in the sarcoplasmic reticulum causing mitochondrial dysfunction, subsequently stressing the endoplasmic reticulum and resulting in cardiomyocyte apoptosis. NF-κB is activated downstream, causing chronic inflammation via dysregulation of the nitric oxide homeostasis resulting in accumulation of Reactive Oxygen Species (ROS), and eventually heart failure. The inhibition of UPS can be reversible (BTZ-reversible binding to β5 and β5i subunits) or irreversible (carfilzomib - irreversible binding to β5 and β5i subunits). The cardiotoxic effects of carfilzomib, mainly congestive heart failure and subclinical left ventricular systolic dysfunction, are well described in the literature [9, 14-16]. There is sparse data about the cardiovascular effects of BTZ.

The Assessment of Proteasome Inhibition for Extending Remissions (APEX) trial [17] was one of the early trials evaluating BTZ versus high dose dexamethasone for relapsed MM. This study did not find a significant increase in the incidence of cardiac events in the BTZ cohort when compared to the dexamethasone cohort (15% vs. 13%, respectively).

A single-center retrospective analysis by Gurram et al. [18] in 2017 did not show an increased incidence of cardiac events in patients treated with BTZ when compared to age-matched controls. They also found that patients who developed cardiac events with BTZ were known to have a higher cardiac risk profile at baseline. A prospective study in 2018 by Heitner et al. [19] evaluated 11 patients on BTZ therapy for the incidence of cardiotoxicity using echocardiography including GLS and CMRI at 3-month intervals during the treatment phase did not find any incidence of cardiotoxicity.

The ENDEAVOR trial [20] evaluated the efficacy and safety of carfilzomib (464 enrolled patients) in direct comparison to BTZ (465 patients) in the treatment of MM. The overall incidence of clinical heart failure was significantly higher in the carfilzomib group compared to the BTZ group (9% vs. 4%). In a subset of the study population who underwent screening echocardiograms, the incidence of left ventricular dysfunction on echocardiogram was similar between the two groups (2 patients in each group). However, they do report that 3 out of the 4 patients who had LV dysfunction recovered on subsequent echocardiograms, although the treatment plan was not mentioned.

A meta-analysis by Xiao et al. [10] in 2014 included 25 clinical trials with 5718 patients evaluated the incidence of cardiotoxicity associated with BTZ. The outcome measures included cardiac arrhythmias, cardiac arrest, cardiomyopathy, congestive heart failure, and decline in LVEF. They did not find a statistically significant increase in the risk of cardiotoxicity among patients treated with BTZ, although there was a 3.8% incidence of all grades and 2.3% incidence of high-grade cardiotoxicity with an overall mortality of 3% in the BTZ group.

There have been several other case reports describing BTZ-induced congestive heart failure [21-24]. The authors do not report the treatment of LV dysfunction with GDMT, although some report improvement in LV function after discontinuation of BTZ. The cumulative doses are reported in 2 studies - 28.66mg/m2 by Gupta et al. [25] and 20. 8mg/m2 by Orciuolo et al [26].

Aside from these, there are isolated case reports of BTZ-induced complete heart block with myocardial scar on CMRI [27-29] requiring permanent pacemaker implantation. There are also cases of BTZ-induced tachyarrhythmias such as supraventricular tachycardia and atrial fibrillation reported in the literature [30]. Nowis et al. performed an animal study [31] that demonstrated cardiotoxicity, cardiomyopathy, and heart failure from BTZ. However, the literature on human models has not established a direct association.

The use of GDMT [32] for left ventricular systolic dysfunction includes treatment with beta-blockers [33, 34], renin-angiotensin-aldosterone system modulation with angiotensin-converting enzyme inhibitors [35]/angiotensin II receptor blockers [36]/angiotensin receptor neprilysin inhibition [37], mineralocorticoid receptor antagonists [38] and sodium-glucose co-transporter 2 inhibitors [39] which have been proven to have a mortality benefit in landmark clinical trials. In addition, there are other classes of medications that are beneficial in certain population groups with LV systolic dysfunction [32]. Traditionally, these same medications have been used for patients with cancer therapeutics related cardiac dysfunction, including anthracycline and HER-2neu receptor blocker-induced cardiotoxicity/LV dysfunction [40, 41].

Patterns of LV dysfunction with BTZ have not been well described since most of these case reports did not have data from CMRI. Foley et al. [42] describe mid-wall late gadolinium enhancement on CMRI in a patient with BTZ-induced LV dysfunction. However, the patient had received prior anthracycline therapy, so the authors concluded that they could not totally attribute the LGE and LV dysfunction to BTZ therapy.

Our patients (2 out 3 had CMRI) did not have any evidence of scar or LGE on CMRI, which supports the hypothesis of myocardial recovery with appropriate GDMT. Furthermore, the third patient was successfully and safely treated for relapsed malignant disease with another PI, which signifies the feasibility of utilizing further non-BTZ PI-based therapy in patients adequately supported with GDMT.

To our knowledge, this is the first series of patients who had recovery of BTZ-induced left ventricular systolic function with appropriate GDMT and subsequent discontinuation of BTZ.

Our group did attempt to titrate GDMT to target doses to maximize the chance of LVEF recovery, however, this was limited by orthostatic hypotension from BTZ induced autonomic neuropathy. This needs special attention as neuropathy is a common side effect with BTZ and other PIs. We did make sure that GDMT was continued even after the recovery of LV function.

Currently, no specific recommendations exist for monitoring for BTZ-related cardiotoxicity with imaging or cardiac biomarkers like cardiac troponin and brain natriuretic peptide. It is unclear if the timing of initiation of GDMT impacts the likelihood of reversibility of BTZ-related cardiomyopathy/ However, we do know that early detection of subclinical cardiotoxicity impacts recovery of LV function in other forms of chemotherapy-induced cardiac dysfunction [43]. Future research investigating the detection of subclinical myocardial dysfunction and the impact of early initiation of GDMT is needed. It is also unclear if initiating cardioprotective medications like beta-blockers and ACE inhibitors/ARB’s at the start of treatment in all patients provides benefit. However, cardiovascular risk factor modification in patients before, during, and after MM treatment is certainly warranted, which is the cornerstone of the cardio-oncology practice.

This case series highlights the potential reversibility of BTZ-related cardiomyopathy with the initiation of GDMT, even in patients with a significant underlying cardiac structural abnormality like non-compaction cardiomyopathy. Future studies should help delineate specific monitoring for cardiotoxicity with BTZ, be it overt or subclinical, as well the outcomes of patients who have recovery in LV function and are re-challenged with BTZ after initial cessation.

## Conclusions

This case series highlights the potential reversibility of BTZ-related cardiomyopathy with the initiation of GDMT, even in patients with a significant underlying cardiac structural abnormality like non-compaction cardiomyopathy. Future studies should help delineate specific monitoring for cardiotoxicity with BTZ, be it overt or subclinical, as well the outcomes of patients who have recovery in LV function and are re-challenged with BTZ after initial cessation.
